# L-type Ca_V_1.2 deletion in the cochlea but not in the brainstem reduces noise vulnerability: implication for Ca_V_1.2-mediated control of cochlear BDNF expression

**DOI:** 10.3389/fnmol.2013.00020

**Published:** 2013-08-09

**Authors:** Annalisa Zuccotti, Sze C. Lee, Dario Campanelli, Wibke Singer, Somisetty V. Satheesh, Tommaso Patriarchi, Hyun-Soon Geisler, Iris Köpschall, Karin Rohbock, Hans G. Nothwang, Jing Hu, Johannes W. Hell, Thomas Schimmang, Lukas Rüttiger, Marlies Knipper

**Affiliations:** ^1^Molecular Physiology of Hearing, Hearing Research Center Tübingen, Department of Otolaryngology, University of TübingenTübingen, Germany; ^2^AG Neurogenetik, Cluster of Excellence “Hearing4all”, Neurogenetics, Carl von Ossietzky Universität OldenburgOldenburg, Germany; ^3^Department of Pharmacology, University of California at DavisDavis, CA, USA; ^4^Centre for Integrative NeuroscienceTübingen, Germany; ^5^Instituto de Biología y Genética Molecular, Departamento de Bioquímica, Biología Molecular y Fisiología, Facultad de Medicina, Universidad de Valladolid y Consejo Superior de Investigaciones CientificasValladolid, Spain

**Keywords:** L-VGCCs, Ca_V_1.2, inner ear, SOC, ABR, BDNF

## Abstract

Voltage-gated L-type Ca^2+^ channels (L-VGCCs) like Ca_V_1.2 are assumed to play a crucial role for controlling release of trophic peptides including brain-derived neurotrophic factor (BDNF). In the inner ear of the adult mouse, besides the well-described L-VGCC Ca_V_1.3, Ca_V_1.2 is also expressed. Due to lethality of constitutive Ca_V_1.2 knock-out mice, the function of this ion channel as well as its putative relationship to BDNF in the auditory system is entirely elusive. We recently described that BDNF plays a differential role for inner hair cell (IHC) vesicles release in normal and traumatized condition. To elucidate a presumptive role of Ca_V_1.2 during this process, two tissue-specific conditional mouse lines were generated. To distinguish the impact of Ca_V_1.2 on the cochlea from that on feedback loops from higher auditory centers Ca_V_1.2 was deleted, in one mouse line, under the Pax2 promoter (Ca_V_1.2^Pax2^) leading to a deletion in the spiral ganglion neurons, dorsal cochlear nucleus, and inferior colliculus. In the second mouse line, the Egr2 promoter was used for deleting Ca_V_1.2 (Ca_V_1.2^Egr2^) in auditory brainstem nuclei. In both mouse lines, normal hearing threshold and equal number of IHC release sites were observed. We found a slight reduction of auditory brainstem response wave I amplitudes in the Ca_V_1.2^Pax2^ mice, but not in the Ca_V_1.2^Egr2^ mice. After noise exposure, Ca_V_1.2^Pax2^ mice had less-pronounced hearing loss that correlated with maintenance of ribbons in IHCs and less reduced activity in auditory nerve fibers, as well as in higher brain centers at supra-threshold sound stimulation. As reduced cochlear BDNF mRNA levels were found in Ca_V_1.2^Pax2^ mice, we suggest that a Ca_V_1.2-dependent step may participate in triggering part of the beneficial and deteriorating effects of cochlear BDNF in intact systems and during noise exposure through a pathway that is independent of Ca_V_1.2 function in efferent circuits.

## INTRODUCTION

Activity-dependent gene transcription is functionally relevant for animals to acquire information and adapt to the environment. One major ion involved in transducing neuronal activity and regulating transcription is calcium (Ca^2+^). Ca^2+^ influx through voltage-gated Ca^2+^ channels (VGCCs) serves as a key transducer coupling changes in cell surface membrane potential with local intracellular Ca^2+^ pathways. Among the L-type VGCCs (L-VGCCs), Ca_V_1.2 is also expressed in the organ of corti (Green et al., 1996; Waka et al., 2003) and the brain (Catterall, 2000; Zuccotti et al., 2011). In the brain, Ca_V_1.2 is assumed to participate in altering synapse efficacy through transcriptional control of brain-derived neurotrophic factor (BDNF; Tabuchi et al., 2000; Zuccotti et al., 2011). The BDNF transcription is highly responsive to neural activity. It is up-regulated by learning (Hall et al., 2000; Lubin et al., 2008), physical exercise (Neeper et al., 1995), and kindling or kainate-induced seizures (Dugich-Djordjevic et al., 1992). The L-VGCCs contribute to the asynchronous release of neurotrophins like BDNF from individual vesicles (Barg et al., 2002; Kolarow et al., 2007), which is most probably Ca_V_1.2 dependent (Kolarow et al., 2007). Accordingly, Ca^2+^ entering the cell, either through Ca_V_1.2 or *N*-methyl-D-aspartate (NMDA) receptors can differentially activate the BDNF promoter IV (Zheng et al., 2011), which controls one of the eight untranslated 5′BDNF exons (exon I-VIII) upstream of a common 3′BDNF exon IX that encodes the BDNF protein (Aid et al., 2007). The BDNF transcribed from exon IV is also expressed in the inner ear (Schimmang et al., 2003) and is changed after ototoxic drug treatment through Ca^2+^-responsive elements such as CaRF1 in cochlear neurons (Singer et al., 2008). It is also found that BDNF is up-regulated following auditory trauma (Tan et al., 2007). These observations may be related to the contrasting roles of BDNF previously described in the intact or injured cochlea (Zuccotti et al., 2012).

On the one side, BDNF plays a crucial role to upgrade complexity of the inner hair cell (IHC) synapse, including maintenance of mature IHC number of synaptic ribbons, electron-dense presynaptic specializations that tether synaptic vesicles for exocytosis at the active zone (Fuchs, 2005; Moser et al., 2006; Schmitz, 2009) in mice (Zuccotti et al., 2012) and zebrafish (Mo and Nicolson, 2011). On the other side, BDNF is harmful when acoustic overstimulation damages the mature system (Zuccotti et al., 2012). As IHC ribbons are crucial for precision of sound processing (Buran et al., 2010) and IHC ribbon loss and deafferentation after acoustic trauma is discussed in the context of age-dependent hearing loss, hyperacusis, and tinnitus (Kujawa and Liberman, 2009; Lin et al., 2011; Singer et al., 2013), the elucidation of the molecular basis of these effects is of crucial clinical relevance. As Ca_V_1.2 (Favereaux et al., 2011) and BDNF up-regulation (Coull et al., 2005; Trang et al., 2011) is also discussed in the context of pain, our findings may contribute to the understanding of sensory pathology beyond the auditory field.

To elucidate to what extent Ca_V_1.2 might participate in the described roles of BDNF in the healthy and injured cochlea, we analyzed Ca_V_1.2 function following conditional deletion of Ca_V_1.2 in the auditory system. This approach is essential as mice globally lacking Ca_V_1.2 die *in utero* before day 15 post-coitum (Seisenberger et al., 2000). We conditionally inactivated Ca_V_1.2 in the auditory system using the same Cre transgenic mouse line as used for deletion of BDNF (Zuccotti et al., 2012). As feedback loops from higher auditory centers, known as the corticofugal pathway (Feliciano and Potashner, 1995), have been shown to modulate directly efferent feedback along the descending auditory pathway as well as the IHC in a frequency-specific way (Xiao and Suga, 2002), we studied mice with an inactivation of Ca_V_1.2 in the superior olivary complex (SOC) that represent the second central auditory processing center in the ascending auditory pathway, using the Cre expression under the Egr2 promoter (Voiculescu et al., 2000; Satheesh et al., 2012).

Both Ca_V_1.2^Pax2^ and Ca_V_1.2^Egr2^ conditional knock-out (KO) mice were viable and thus could be analyzed for hearing capability, sound processing along the ascending auditory pathway, and sensitivity to noise exposure. We demonstrate here that Ca_V_1.2 is neither required in the cochlea nor in the SOC for normal IHC or outer hair cell (OHC) function, thus suggesting that BDNF function in normal IHC physiology (Zuccotti et al., 2012) does not depend on Ca_V_1.2. In contrast, loss of Ca_V_1.2 in the cochlea, but not in the SOC, partially mimics the described harmful BDNF effect during acoustic trauma, a feature that could be linked to reduced BDNF mRNA levels in cochlear tissue of Ca_V_1.2^Pax2^ mice. The results are discussed in the context of a role of L-type Ca^2+^ channels for BDNF release during cochlear injury.

## MATERIALS AND METHODS

Care and use of animals and the experimental protocol were reviewed and approved by animal welfare commissioner and the regional board for scientific animal experiments in Tübingen, Germany.

### GENERATION OF CONDITIONAL KNOCK-OUT MICE

The conditional Ca_V_1.2 knock-out mice Ca_V_1.2^Pax2^ KO were generated by breeding mice carrying the floxed Ca_V_1.2 allele (L2; Seisenberger et al., 2000) with mice expressing Cre under control of Pax2 regulatory regions (Ohyama and Groves, 2004) carrying one floxed Ca_V_1.2 allele (L1; Seisenberger et al., 2000), resulting in cochlea-specific Ca_V_1.2^Pax2^ KO mice (Ca_V_1.2 L1/L2, Pax2::Cre) and control animals (Ca_V_1.2+/L2, Pax2::Cre). Besides the cochlea, Ca_V_1.2 was deleted in the dorsal cochlear nucleus (DCN), inferior colliculus (IC), and the cerebellum in these mice (Ohyama and Groves, 2004; Zuccotti et al., 2012).

The Ca_V_1.2^Egr2^ KO mice were generated by breeding mice carrying floxed Ca_V_1.2 alleles (L2; Seisenberger et al., 2000) with mice carrying one Ca_V_1.2 KO allele (L1; Seisenberger et al., 2000) and expressing Cre from one allele of the Egr2 locus (Voiculescu et al., 2000), which mediates recombination in rhombomeres 3 and 5 of the embryonic neural tube, allowing genetic manipulation of neuronal populations such as the lateral superior olive (LSO) and medial nucleus of the trapezoid body (MNTB) but not in the cochlea, obtaining a specific knock-out in the LSO and MNTB (Voiculescu et al., 2000; Rosengauer et al., 2012; Satheesh et al., 2012).

### HEARING MEASUREMENTS: AUDITORY BRAINSTEM RESPONSE AND DISTORTION PRODUCT OTOACOUSTIC EMISSION

Auditory brainstem response (ABR), evoked by short-duration sound stimuli, represents the summed activity of neurons in distinct anatomical structures or nuclei along the ascending auditory pathway (Burkard and Don, 2007) and is measured by averaging the evoked electrical response recorded *via* subcutaneous electrodes. The ABR to click and pure tone stimuli and the cubic 2 × *f*1 - *f*2 distortion product of the otoacoustic emission (DPOAE) for *f*2 = 1.24 × *f*1 and L2 = L1 - 10 dB were recorded in anesthetized mice aged 6–9 weeks. All physiological recordings were performed under anesthesia [75 mg/kg ketamine hydrochloride (Ketavet, Pharmacia, Pfitzer, Karlsruhe, Germany), 5 mg/kg xylazine hydrochloride (Rompun 2%, Bayer Leverkusen, Germany), 0.2 mg/kg atropine (Atropinsulfat B. Braun, Melsungen, Germany)] in a soundproof chamber (IAC, Niederkrüchten, Germany) as previously described (Engel et al., 2006). In short, ABR thresholds were determined with click (100 μs), and pure tone (2–45.3 kHz, 3 ms duration) stimuli. OHC function was assessed by the DPOAE thresholds evoked by stimuli at various frequencies (*f*2 = 4–32 kHz with half-octave steps) and growth function of the DPOAE evoked by *f*2 = 11.3 kHz.

### NOISE EXPOSURE

For noise exposure, animals were exposed to broadband noise (4–16 kHz, 120 dB sound pressure level (SPL) for 1 h) as previously described (Engel et al., 2006) while under anesthesia (see above), and supplemental doses of anesthetics were administered subcutaneously every 20 min. Sham-exposed animals were anesthetized and placed in the reverberating chamber, but not exposed to acoustic stimulus (i.e., the speaker remained turned off). In noise-exposed mice, the degree of the ABR threshold shift was measured more than 7 days after noise exposure, when noise-induced permanent threshold shift (PTS; Liberman, 1980; Salvi et al., 1986) have settled and a recovery from damage is no longer expected. The sham-exposed animals have completely normal hearing.

### TISSUE PREPARATION

For immunohistochemistry, cochleae were isolated, fixed by immersion in 2% paraformaldehyde, 125 mM sucrose in 100 mM phosphate-buffered saline, pH 7.4, for 2 h, and then decalcified for 45 min in RDO rapid decalcifier (Apex Engineering Products Corporation, Aurora, IL, USA) as previously described (Knipper et al., 1999; Zuccotti et al., 2012; Singer et al., 2013), cryosectioned at 10 μm, mounted on SuperFrost*/plus microscope slides at -20°C.

For RNA and proteins isolation, cochleae and different brain regions were dissected with small forceps and immediately frozen in liquid nitrogen and stored at -80°C until use.

### RNA ISOLATION, cDNA SYNTHESIS, AND REAL-TIME PCR

For real-time polymerase chain reaction (PCR) analysis, RNA from mouse cochlea was isolated using the RNeasy mini kit (QIAGEN, Hilden, Germany) following the manufacturer’s instructions. After reverse transcription using Sensiscript RT kit (QIAGEN), real-time PCR was performed using the iCycler iQ detection system (Bio-Rad, Munich, Germany). The real-time PCR reaction was set up following the manufacturer’s instruction (QuantiFast SYBR Green, QIAGEN). The following primers were used: BDNF quantification, for 5′-GAAGCAAACGTCCACGGACAA-3′ and rev 5′-AACCTTCTGGTCCTCATCCAG-3′; β-actin quantification, used as housekeeping gene, for 5′-GAATCCTGTGGCATCCATGA-3′ and rev 5′-CATCTGCTGGAAGGTGGACA-3′. The PCR program, according to the manufacturer’s instructions, included an initial activation step at 95°C for 5 min, followed by 40 cycles of a 10-s lasting denaturing step at 95°C and a 30-s combined annealing/extension step at 60°C; all temperature transition rates were 1–3°C/s. At the end of each cycle, the fluorescence emitted by SYBR Green was measured. At the end of the PCR reaction, samples were subjected to a temperature ramp (from 70 to 95°C, 2°C/s) with continuous fluorescence monitoring for melting curve analysis. For each PCR product, a single narrow peak was obtained by melting curve analysis at the specific temperature. Each sample was assayed in triplicate and the analysis was performed with Gene Expression Macro (version 1.1, Bio-Rad, Munich, Germany). Samples containing no template were used as negative controls in each experiment. Data were normalized to the housekeeping gene expression level and presented as a percentage of the expression relative to the control ± standard deviation (SD).

### IMMUNOHISTOCHEMISTRY

For immunohistochemistry, mouse cochlear sections were stained as previously described (Tan et al., 2007; Zuccotti et al., 2012; Singer et al., 2013). The CtBP2/RIBEYE, mouse (BD Transduction Laboratories, USA); Otoferlin, rabbit (Schug et al., 2006); and Ca_V_1.2, rb (Hell et al., 1993) were used as primary antibodies. For double labeling studies, both antibodies were simultaneously incubated for identical time periods. Sections were viewed, as previously described (Zampini et al., 2010), using an Olympus BX61 microscope equipped with epifluorescence illumination. Images were acquired using an Olympus XM10 CCD monochrome camera and analyzed with cellSens Dimension software (OSIS GmbH, Munster, Germany). To increase spatial resolution, slices were imaged over a distance of 15 μm within an image-stack along the *z*-axis (*z*-stack) followed by 3-dimensional deconvolution using cellSens dimension built-in algorithm.

### NORTHERN BLOT

The mRNA isolation was performed using the Oligotex Direct mRNA Mini Kit (QIAGEN). The mRNA was loaded onto a denaturing 0.8% agarose formaldehyde gel and transferred onto a nylon membrane (Roche, Mannheim, Germany). The membrane was blocked for 30 min at 65°C with hybridization buffer, digoxigenin Easy Hyb (Roche), and hybridized overnight at 65°C with riboprobes for BDNF and cyclophilin. After washing, a 1-h blocking step was performed. The membrane was incubated with anti-Dig-AP (Roche; 1:20,000). The mRNA was detected with CDP-Star ready-to-use (Roche) and exposed to X-ray films.

### WESTERN BLOT

Western blot with the antibody CNC1, which is directed against Loop II/III of α_1_ 1.2, the central Ca_V_1.2 subunit, was performed as previously described (Hell et al., 1993; Davare et al., 1999). Briefly, tissue samples were extracted with 3× sodium dodecyl sulfate-polyacrylamide gel electrophoresis (SDS-PAGE) sample buffer at 90°C for 5 min before SDS-PAGE in 7.5% polyacrylamide gels, transfer to polyvinylidene difluoride (PVDF) membranes, blocking with 10% milk powder and detection with CNC1 and horseradish peroxidase-coupled protein A. Tubulin with an anti-tubulin monoclonal mouse antibody (Santa Cruz sc-5286, Dallas, TX, USA) was detected in parallel at the lower part of the blot to ensure equal loading when indicated.

### DATA ANALYSIS

#### ABR waveform amplitude analysis

The ABR waveforms were analyzed for consecutive amplitude deflections (waves), each wave consisting of a starting negative (n) peak and the following positive (p) peak. Peak amplitudes of ABR waves I and III were extracted in the present study and defined as follows: wave I: I_n_ - I_p_ (0.9–2 ms), wave III: III_n_ - III_p_ (3.3–4.1 ms). A customized program was used for the extraction of ABR peaks based on the definitions given above. ABR peak-to-peak, or wave amplitude growth functions were constructed for individual ears based on the extracted peaks for increasing stimulus levels. All ABR wave amplitude growth functions were calculated for increasing stimulus levels with reference to the ABR thresholds (from -20 to a maximum of 75 dB above threshold before noise exposure and from -20 to a maximum of 55 dB above threshold after noise exposure). For illustration purpose, ABR wave amplitude growth functions as shown in **Figures 3A,B,D,E** and **6A,B** were first linearly interpolated to the resolution of 1 data point/dB and then smoothed by a moving zero-phase Gaussian filter with a window length of 9 data points. The ABR waveforms shown in the inset of the figures were smoothed by a moving zero-phase Gaussian filter with a window length of 5 data points (0.5 ms).

#### Statistical analysis

Unless otherwise stated, all data were presented as group mean with SD or with standard error of the mean (SEM). Differences of the means were compared for statistical significance either by Student’s *t*-test, one-way, or two-way ANOVA tests. The ANOVA tests were followed by multiple *t*-tests with Bonferroni–Holm’s adjustment of α levels. Statistical significance was tested at the level α = 0.05, and resulting *p* values are reported in the legends. *p < 0.05; **p < 0.01; ***p < 0.001; n.s., not significant.

## RESULTS

### CONDITIONAL DELETION OF Ca_V_1.2 DRIVEN BY THE Pax2 AND Egr2 PROMOTERS

To circumvent embryonic lethality of mice globally lacking Ca_V_1.2, Ca_V_1.2 loxP/loxP (or floxed) mice (Seisenberger et al., 2000) were either mated to Pax2::Cre mice (Ohyama and Groves, 2004) or to Egr2::Cre mice (Voiculescu et al., 2000).

**FIGURE 1:**
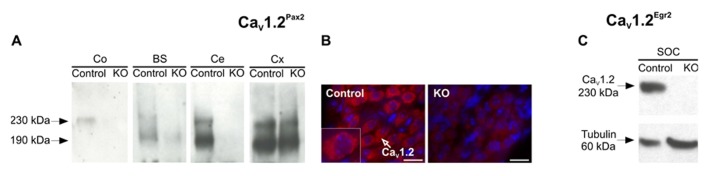
**Tissue-specific deletion of Ca_V_1. 2 in Ca_V_1.2^Pax2^ KO and Ca_V_1.2^Egr2^ KO mice.** **(A)** Western blot for Ca_V_1.2 protein (230 and 190 kDa proteolytic product) in mouse cochlea (Co), brainstem (BS), cerebellum (Ce), and cortex (Cx) from control and Ca_V_1.2^Pax2^ KO mice. No Ca_V_1.2 protein was detected in tissues from KO mice, with the exception for the cortex where the Cre-mediated recombination does not occur [*n* = 10 cochleae (5 mice), 2 brainstem (2 mice), 2 cerebella (2 mice), 2 auditory cortex (2 mice); experiments done in triplicate]. **(B)** Immunofluorescent labeling of Ca_V_1.2 in spiral ganglion neurons from control mice (left), shown at higher magnification (insert) and Ca_V_1.2^Pax2^ KO mice (right) using an antibody against Ca_V_1.2 (red). Nuclei were counterstained with 4′,6-diamidino-2-phenylindole (DAPI; blue). **(C)** Western blot for Ca_V_1.2 protein (230 kDa) and tubulin (60 kDa) in control and Ca_V_1.2^Egr2^ KO mice confirming the deletion of Ca_V_1.2 in the superior olivary complex (SOC; *n* = 3 control mice, 3 KO mice; experiments done in triplicate).

As predicted for the usage of Cre under control of the Pax2 promoter (Ohyama and Groves, 2004; Zuccotti et al., 2012), Ca_V_1.2^Pax2^ KO mice exhibited a deletion of Ca_V_1.2 in the cochlea, brainstem, and cerebellum, but not in the auditory cortex. This was detected by Western blot analysis [**Figure 1A**, *n* = 10 cochleae (5 mice), 2 brainstem (2 mice), 2 cerebella (2 mice), 2 auditory cortex (2 mice); experiments done in triplicate]. The deletion of Ca_V_1.2 in spiral ganglion neurons (SGNs; Warr and Guinan, 1979) was confirmed by immunohistochemistry (**Figure 1B**, Ca_V_1.2, red). No Ca_V_1.2 staining was observed in Ca_V_1.2^Pax2^ KO animals in comparison to control littermates (**Figure 1B**, red).

Usage of Cre under the control of the Egr2 promoter leads to a knock-out of Ca_V_1.2 in the SOC forming part of the auditory brainstem (Voiculescu et al., 2000; Rosengauer et al., 2012; Satheesh et al., 2012). This was confirmed by Western blot of the SOC (*n* = 3 control mice, 3 KO mice; experiments done in triplicate) where no Ca_V_1.2 expression was detected (**Figure 1C**).

### HEARING FUNCTION IN Ca_V_1.2 MOUSE LINES

To test whether the lack of Ca_V_1.2 in the cochlea or brainstem affects hearing function at adult stages, ABR and DPOAE in 2- to 4-month-old mice were measured.

**FIGURE 2:**
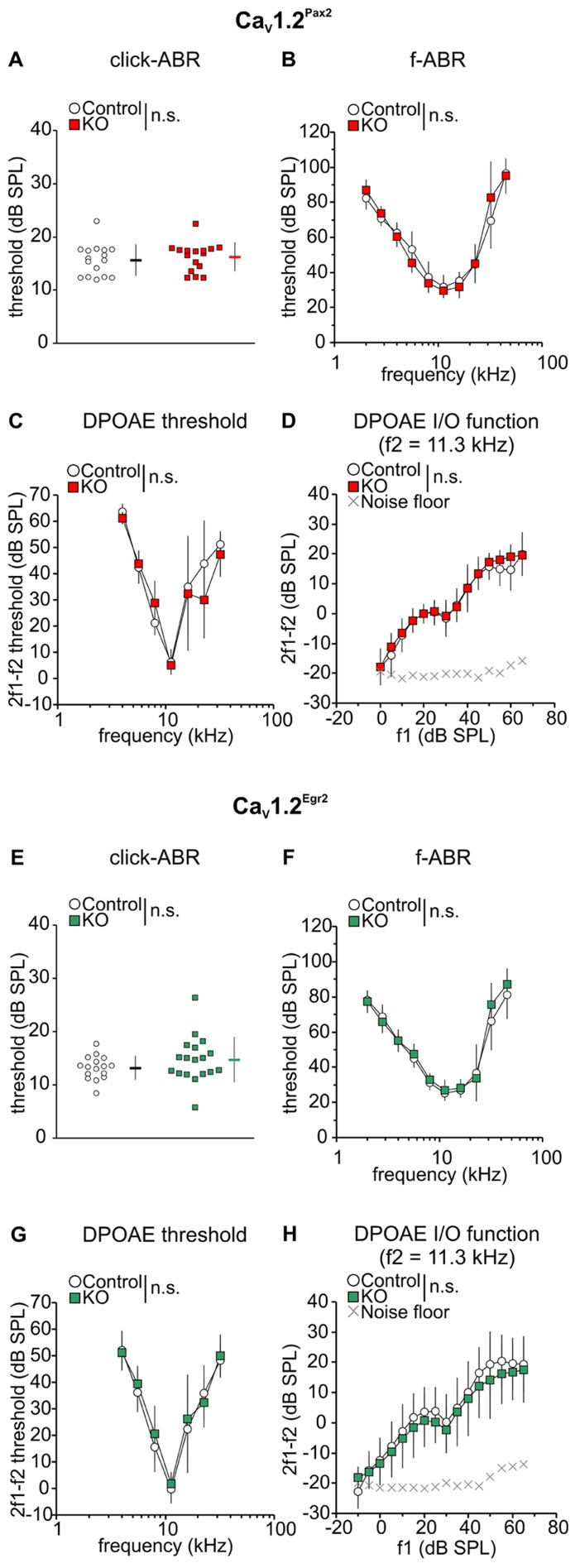
**No deterioration of ABR thresholds and OHC function in Ca_V_1.2^Pax2^ KO and Ca_V_1.2^Egr2^ KO mice.** **(A)** Mean ± SD click-evoked ABR (click-ABR) thresholds for Ca_V_1.2^Pax2^ control (black horizontal dash, single ear thresholds as white circles) and Ca_V_1.2^Pax2^ KO mice (red horizontal dash, single ear thresholds as red squares). Thresholds were not significantly different (two-sided Student’s *t*-test: *p* = 0.556) between KO mice (16.2 ± 2.71, *n* = 16 ears, 8 mice) and control (15.6 ± 2.97, *n* = 16 ears, 8 mice). **(B)** Mean ± SD tone-burst-evoked ABR (f-ABR) thresholds for Ca_V_1.2^Pax2^ KO mice (red) and controls (white). Thresholds were not significantly different (two-way ANOVA, *p* = 0.890) between KO mice (*n* = 6–8 ears, 6–8 mice) and controls (*n* = 7–8 ears, 7–8 mice). **(C)** Mean ± SD DPOAE thresholds for Ca_V_1.2^Pax2^ control mice (control, white) and Ca_V_1.2^Pax2^ KO mice (KO, red). Thresholds were not significantly different (two-way ANOVA: *p* = 0.452) between KO mice (*n* = 4 ears, 4 mice) and controls (*n* = 4 ears, 4 mice). **(D)** Mean ± SD DPOAE amplitude I/O function evoked by stimulus f2 = 11.3 kHz for Ca_V_1.2^Pax2^ KO mice (red) and controls (white) showing no significant difference (two-way ANOVA: *p* = 0.158) between KO mice (*n* = 4 ears, 4 mice) and controls (*n* = 4 ears, 4 mice). **(E)** Mean ± SD click-ABR thresholds for Ca_V_1.2^Egr2^ control mice (black horizontal dash, single ear thresholds as white circles) and Ca_V_1.2^Egr2^ KO (green horizontal dash, single ear thresholds as green squares). Thresholds were not significantly different (two-sided Student’s *t*-test: *p* = 0.204) between KO mice (14.7 ± 4.28, *n* = 18 ears, 9 mice) and controls (13.2 ± 2.20, *n* = 16 ears, 8 mice). **(F)** Mean ± SD f-ABR thresholds for Ca_V_1.2^Egr2^ KO mice (green) and controls (white). Thresholds were not significantly different (two-way ANOVA, *p* = 0.132) between KO mice (*n* = 13 ears, 9 mice) and controls (*n* = 12–13 ears, 8–9 mice). **(G)** Mean ± SD DPOAE thresholds for Ca_V_1.2^Egr2^ KO mice (green) and controls (Ca_V_1.2^Egr2^ control, white). Thresholds were not significantly different (two-way ANOVA: *p* = 0.195) between KO mice (*n* = 16–18 ears, 9 mice) and controls (*n* = 14–15 ears, 8 mice). **(H)** Mean ± SD DPOAE amplitude I/O function evoked by stimulus f2 = 11.3 kHz for Ca_V_1.2^Egr2^ KO mice (green) and controls (white) showing no significant difference (two-way ANOVA: *p* = 0.566) between KO mice (*n* = 16–18 ears, 9 mice) and controls (*n* = 14–15 ears, 8 mice).

For the Ca_V_1.2^Pax2^ mice, no significant difference was observed compared to controls for click-evoked ABR thresholds (**Figure 2A**, Ca_V_1.2^Pax2^ control: 15.6 ± 2.97 dB SPL; Ca_V_1.2^Pax2^ KO: 16.2 ± 2.71 dB SPL; *n* = 16 ears, 8 mice each, two-sided Student’s *t*-test: *p* = 0.556) or tone-burst evoked ABR thresholds (**Figure 2B**, Ca_V_1.2^Pax2^ control: *n* = 7–8 ears, 7–8 mice; Ca_V_1.2^Pax2^ KO: *n* = 6–8 ears, 6–8 mice, two-way ANOVA, *p* = 0.890). Similar results were obtained for the Ca_V_1.2^Egr2^ mouse line for click-evoked stimuli (**Figure 2E**, Ca_V_1.2^Egr2^ control: 13.2 ± 2.20, *n* = 16 ears, 8 mice; Ca_V_1.2^Egr2^ KO: 14.7 ± 4.28, *n* = 18 ears, 9 mice, two-sided Student’s *t*-test: *p* = 0.204) and tone-burst evoked ABR thresholds (**Figure 2F**, Ca_V_1.2^Egr2^ control: *n* = 12–13 ears, 8–9 mice; Ca_V_1.2^Egr2^ KO: *n* = 13 ears, 9 mice, two-way ANOVA, *p* = 0.132).

Since OHC electromotility codetermines the sound-evoked neural potentials at threshold (El-Badry and McFadden, 2007), DPOAE were measured as an objective indicator of OHC function. The DPOAE thresholds (**Figures 2C,G**) and the input/output (I/O) function of emission amplitudes evoked by stimulus frequency of 11.3 kHz (**Figures 2D,H**), the frequency showing the best hearing sensitivity for the mice, were found to be similar between control and Ca_V_1.2^Pax2^ KO mice (Ca_V_1.2^Pax2^ control: *n* = 4 ears, 4 mice; Ca_V_1.2^Pax2^ KO: *n* = 4 ears, 4 mice, two-way ANOVA, *p* = 0.452) and Ca_V_1.2^Egr2^ KO (Ca_V_1.2^Egr2^ control: *n* = 14–15 ears, 8 mice; Ca_V_1.2^Egr2^ KO: *n* = 16–18 ears, 9 mice), indicating that loss of Ca_V_1.2 did not influence motility of OHCs.

**FIGURE 3:**
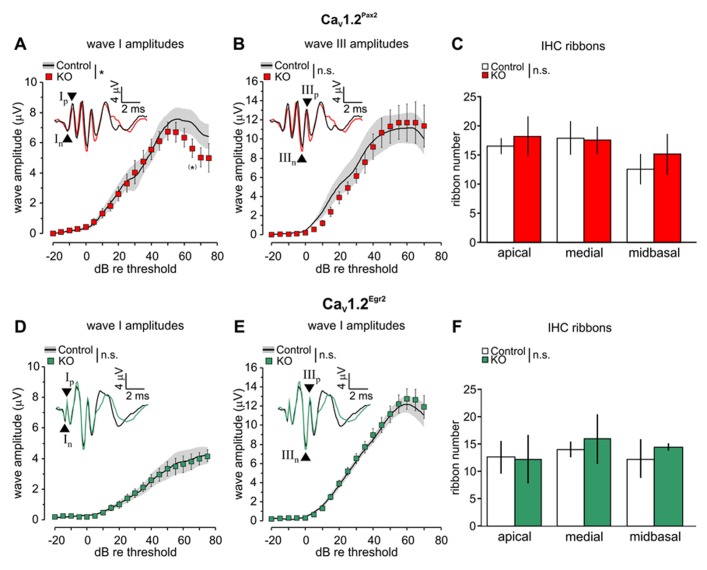
**Reduction of ABR wave I amplitudes and unaltered ABR wave III amplitudes in Ca_V_1.2^Pax2^ KO mice.** **(A)** Mean ± SEM click-evoked ABR wave I amplitude growth function for Ca_V_1.2^Pax2^ control (control, black line, and gray area) and Ca_V_1.2^Pax2^ KO mice (KO, red squares). ABR wave I amplitudes were significantly different (two-way ANOVA, *p* = 0.048) between KO (*n* = 5–9 ears, 3–6 mice) and controls (*n* = 3–6 ears, 3–6 mice), particularly at 65 dB above threshold (*post hoc* one-sided Student’s *t*-test: *p* = 0.049, no Bonferroni–Holm’s adjustment for multiple testing). The black and red waveforms in the inset depict representative ABR traces measured from one individual ear of a Ca_V_1.2^Pax2^ control and a KO mouse, respectively, evoked at the level of 65 dB above the hearing threshold. The leading negative (n) and following positive (p) peak corresponded to wave I are indicated by an upward and a downward pointing arrow head associated with symbols “I_n_” and “I_p_” respectively. **(B)** Mean ± SEM click-evoked ABR wave III amplitude growth function for controls and Ca_V_1.2^Pax2^ KO mice. ABR wave III amplitudes were not significantly different (two-way ANOVA, *p* = 0.639) between KO (*n* = 2–5 ears, 2–4 mice) and controls (*n* = 3 ears, 2 mice). The leading negative (n) and following positive (p) peak corresponded to wave III are indicated by arrow heads and marked “III_n_” and “III_p_” respectively. **(C)** Mean synaptic ribbon number ± SD are not significantly different (two-way ANOVA, *p* = 0.260) between control and Ca_V_1.2^Pax2^ KO mice in apical, medial, and midbasal cochlear turns (control: *n* = 4 cochleae, 3 mice; KO: *n* = 3 cochleae, 3 mice). **(D) **Mean ± SEM click-evoked ABR wave I amplitude growth function for Ca_V_1.2^Egr2^ KO mice (green squares) and controls (Ca_V_1.2^Egr2^ control, black and gray area). ABR wave I amplitudes were not significantly different (two-way ANOVA, *p* = 0.784) between KO (*n* = 7–11 ears, 4–8 mice) and controls (*n* = 12–13 ears, 7 mice). The black and green waveforms in the inset depict representative ABR traces measured from one individual ear of a Ca_V_1.2^Egr2^ control mouse and KO mouse, respectively, evoked at the level of 65 dB above the hearing threshold. **(E)** Mean ± SEM click-evoked ABR wave III amplitude growth function for Ca_V_1.2^Egr2^ KO mice and controls. ABR wave III amplitudes were not significantly different (two-way ANOVA, *p* = 0.281) between KO (*n* = 9–12 ears, 5–7 mice) and controls (*n* = 10–11 ears, 6 mice). **(F)** Mean ± SD IHC ribbon counts for Ca_V_1.2^Pax2^ control (control, white bars) and Ca_V_1.2^Pax2^ KO mice (KO, green bars) in apical, medial, and midbasal cochlear turns. No significant difference (two-way ANOVA, *p* = 0.446) between the number of ribbons in control and Ca_V_1.2^Egr2^ KO mice (control: *n* = 3 cochleae, 3 mice; KO: *n* = 3 cochleae, 3 mice).

The click-evoked ABR waveform amplitudes are expected to change proportionally to the size of discharge rates and number of synchronously firing auditory nerve (AN) fibers (Johnson and Kiang, 1976). We, therefore, analyzed ABR wave I (**Figures 3A,D**), reflecting the summed activity of the AN fibers, and ABR wave III (**Figures 3B,E**), corresponding to the SOC (Melcher et al., 1996), in the two Ca_V_1.2 mutant mouse lines where Ca_V_1.2 is missing either in the cochlea or in parts of the auditory brainstem (**Figure 1**). In Ca_V_1.2^Pax2^ mice, but not in Ca_V_1.2^Egr2^ mice, a stronger decline of ABR wave I was observed beginning at 50 dB above threshold (**Figure 3A**, Ca_V_1.2^Pax2^ control: *n* = 3–6 ears, 3–6 mice; Ca_V_1.2^Pax2^ KO: *n* = 5–9 ears, 3–6 mice; two-way ANOVA, *p* = 0.048, *post hoc* one-sided Student’s *t*-test: *p* = 0.049 at 65 dB above threshold, no Bonferroni–Holm’s adjustment for multiple testing; **Figure 3D**, Ca_V_1.2^Egr2^ control: *n* = 12–13 ears, 7 mice; Ca_V_1.2^Egr2^ KO: *n* = 7–11 ears, 4–8 mice; two-way ANOVA: *p* = 0.784). No significant differences were observed for the amplitudes of ABR wave III between KO mice (both Ca_V_1.2^Pax2^ KO and Ca_V_1.2^Egr2^ KO) and the respective controls (**Figures 3B,E**).

Synaptic ribbons are known to be an accurate metric of the afferent innervation of IHCs (Kujawa and Liberman, 2009) that drive postsynaptic AN fibers by a large releasable transmitter pool (Matthews and Fuchs, 2010). The number of synaptic ribbons in the Ca_V_1.2^Pax2^ and Ca_V_1.2^Egr2^ mouse lines (**Figures 3C,F**) was not significantly different observed along the different cochlear turns [apical: 2–7 kHz, medial: 7–16 kHz, and midbasal: >17 kHz (Müller, 1991)] between the mutant lines and their respective controls (**Figure 3C**, control: *n* = 4 ears, 3 mice and Ca_V_1.2^Pax2^ KO: *n* = 3 ears, 3 mice, two-way ANOVA: *p* = 0.260; **Figure 3F**, controls: *n* = 3 ears, 3 mice, Ca_V_1.2^Egr2^ KO: *n* = 3 ears, 3 mice, two-way ANOVA: *p* = 0.446).

### ACOUSTIC TRAUMA EFFECT ON HEARING FUNCTION IN CONDITIONAL Ca_V_1.2 MUTANTS

**FIGURE 4:**
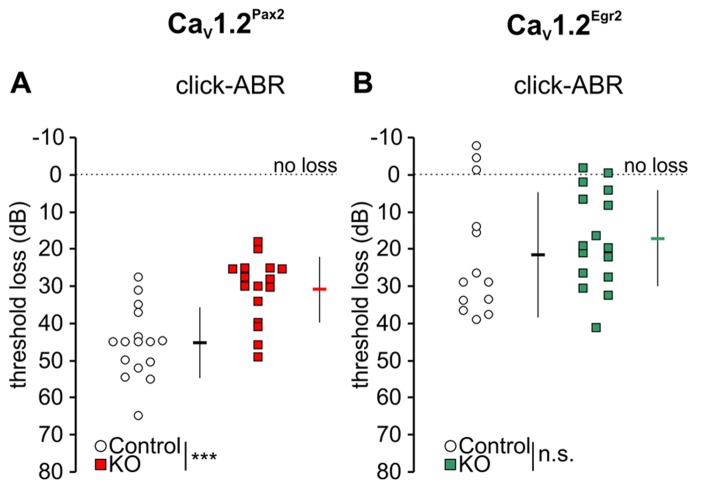
**Mild loss of click-ABR threshold in Ca_V_1.2^Pax2^ KO mice after noise exposure.** **(A)** Mean ± SD click-ABR threshold losses for Ca_V_1.2^Pax2^ controls (black horizontal dash, 45.3 ± 9.51, *n* = 16 ears, 8 mice, single ear threshold losses as white circles) and Ca_V_1.2^Pax2^ KO mice (red horizontal dash, 30.9 ± 8.87, *n* = 16 ears, 8 mice, single ear threshold losses as red squares) 7 days after noise exposure showing a significantly milder threshold loss (14.5 dB difference; two-sided Student’s *t*-test: *p* < 0.001) in Ca_V_1.2^Pax2^ KO mice. **(B)** Mean ± SD click-ABR threshold losses for Ca_V_1.2^Egr2^ controls (black horizontal dash, 21.6 ± 16.8, *n* = 13 ears, 7 mice, single ear threshold losses as white circles) and Ca_V_1.2^Egr2^ KO mice (green horizontal dash, 17.2 ± 12.9, *n* = 16 ears, 8 mice, single ear threshold losses as green squares) 7–11 days after noise exposure, showing similar threshold loss (4.4 dB difference; two-sided Student’s *t*-test: *p* = 0.431). Dotted lines at 0 dB represent no threshold loss after noise exposure.

We studied a possible role of Ca_V_1.2 during noise damage by comparing ABR thresholds between control and Ca_V_1.2^Pax2^ KO and Ca_V_1.2^Egr2^ KO mice after exposing them for 1 h to broadband noise (4–16 kHz) of 120 dB SPL. Hearing thresholds were analyzed by ABR to click stimulus 7–11 days after noise exposure (**Figure 4**).

All noise-exposed animals (controls and KOs) showed significant ABR threshold losses for click stimuli (**Figures 4A,B**; **Tables 1** and **2**).

**Table 1:** Auditory brainstem response thresholds for Ca_V_1.2^Pax2^ mouse line evoked by click stimuli.

	Click-ABR threshold (dB SPL)		Significance
	Pre noise	7 days post noise	
Ca_V_1.2^Pax2^ control	15.6 ± 2.97 (16/8)	60.9 ± 8.90 (16/8)	***
Ca_V_1.2^Pax2^ KO	16.2 ± 2.71 (16/8)	47.1 ± 8.84 (16/8)	***
Significance	n.s.	***	

Mean ± SD (no. of ears/no. of mice); ***p < 0.001 (two-sided *t*-test); n.s., not significant.

**Table 2:** Auditory brainstem response thresholds for Ca_V_1.2^Egr2^ mouse line evoked by click stimuli.

	Click-ABR threshold (dB SPL)		Significance
	Pre noise	7–11 days post noise	
Ca_V_1.2^Egr2^ control	13.2 ± 2.20 (16/8)	34.8 ± 15.70 (13/7)	***
Ca_V_1.2^Egr2^ KO	14.7 ± 4.28 (18/9)	31.9 ± 12.80 (16/8)	***
Significance	n.s.	n.s.	

Mean ± SD (no. of ears/no. of mice); ***p < 0.001 (two-sided *t*-test); n.s., not significant.

**FIGURE 5:**
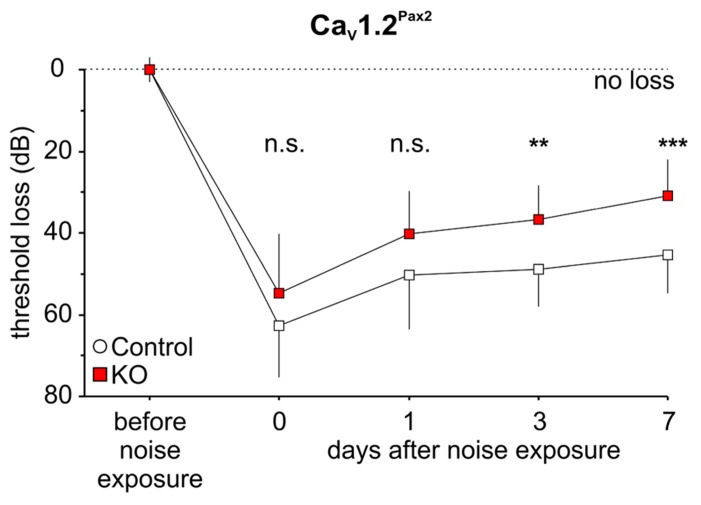
**Preservation of click-ABR thresholds in Ca_V_1.2^Pax2^ KO mice after noise exposure.** Mean ± SD click-ABR threshold losses for control and Ca_V_1.2^Pax2^ KO mice before noise exposure, after noise exposure at the same day, 1 day, 3 days, and 7 days, showing a significant differences in threshold losses (two-way ANOVA, *p* < 0.001) between control (14–16 ears, 7–8 mice) and KO (16 ears, 8 mice). Significant differences in threshold loss were observed at 3 days (*post hoc* two-sided Student’s *t*-test: *p* = 0.007, Bonferroni–Holm’s adjustment for multiple testing) and 7 days (*post hoc* two-sided Student’s *t*-test: *p* < 0.001, Bonferroni–Holm’s adjustment for multiple testing) after noise exposure.

However, ABR thresholds to click stimuli were significantly lower in noise-exposed Ca_V_1.2^Pax2^ KO mice in comparison to noise-exposed controls after 7 days (**Figure 4A**; **Table 1**, comparison between genotypes at 7 days post noise). On the contrary, no differences were observed between control and Ca_V_1.2^Egr2^ KO mice. Reduced vulnerability of click-ABR threshold loss appeared instantaneously and was observed already at the first day after exposure (**Figure 5**), indicating that Ca_V_1.2 deletion may control a key event for threshold loss already shortly after the induction of trauma.

These data suggest that Ca_V_1.2 deletion in the cochlea, but not in the brainstem, protects against noise.

**FIGURE 6:**
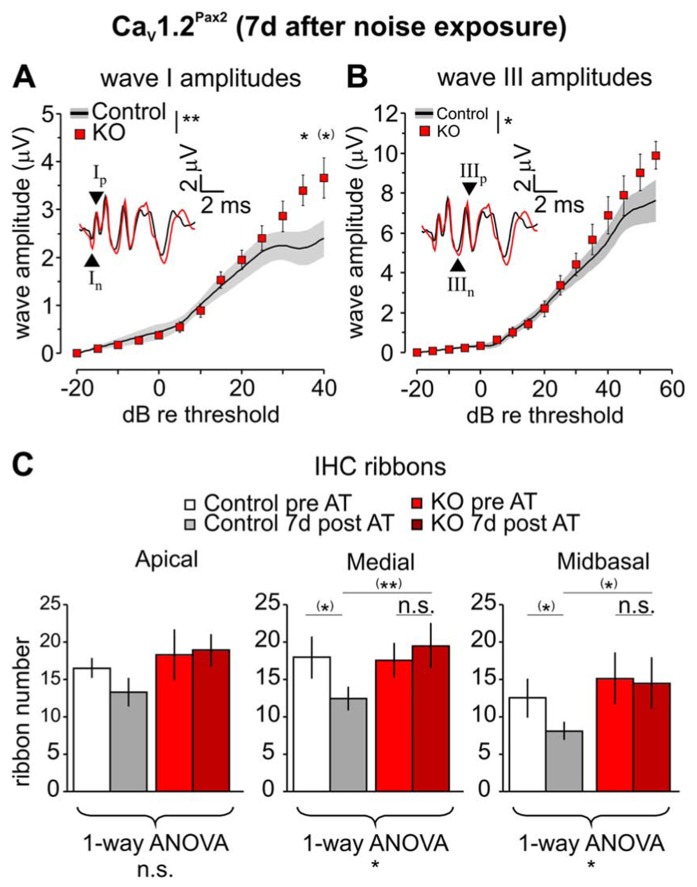
**Less reduction of ABR wave I and wave III amplitudes in Ca_V_1.2^Pax2^ KO mice after noise exposure.** **(A)** Mean ± SEM click-evoked ABR wave I amplitude growth function for Ca_V_1.2^Pax2^ KO mice (red squares) and controls (control, black line and gray area) 7 days after noise exposure. ABR wave I amplitudes were significantly different (two-way ANOVA, *p* = 0.004) between KOs (*n* = 6–13 ears, 5–8 mice) and controls (*n* = 5–11 ears, 5–8 mice), particularly at 35 dB (*post hoc* one-sided Student’s *t*-test: *p* = 0.038, Bonferroni–Holm’s adjustment for multiple testing) and 40 dB (*post hoc* one-sided Student’s *t*-test: *p* = 0.026, no Bonferroni–Holm’s adjustment for multiple testing) above threshold. The black and red waveforms in the inset depict ABR traces measured from one individual ear of a Ca_V_1.2^Pax2^ control and KO mouse, respectively, evoked at the level of 40 dB above the hearing threshold. The leading negative (n) and following positive (p) peak corresponded to wave I are indicated by arrow heads and marked “I_n_” and “I_p_” respectively. **(B)** Mean ± SEM click-evoked ABR wave III amplitude growth function for Ca_V_1.2^Pax2^ KO mice and controls. ABR wave III amplitudes were significantly different (two-way ANOVA, *p* = 0.013) between KOs (*n* = 3–7 ears, 3–4 mice) and controls (*n* = 3–10 ears, 3–6 mice). The leading negative (n) and following positive (p) peak corresponded to wave III are indicated by arrow heads and marked “III_n_” and “III_p_” respectively. **(C)** Mean ribbon counts ± SD of control and Ca_V_1.2^Pax2^ KO before and 7 days after noise exposure showing reduction of the number of ribbons between control mice before and after noise exposure at all three cochlear turns, and was statistically significant at the medial and the midbasal turns (one-way ANOVA, apical turn *p* = 0.060, medial turn *p* = 0.026, midbasal turn *p* = 0.047). However, reduction is mainly observed in the control mice (before noise exposure: white bars, *n* = 4 cochleae, 4 mice; after noise exposure: gray bars, *n* = 3 cochleae, 3 mice; *post hoc* two-sided Student’s *t*-test with no Bonferroni–Holm’s adjustment for multiple testing for medial turn: *p* = 0.018 and midbasal turn: *p* = 0.045) but not in the Ca_V_1.2^Pax2^ KO mice (before noise exposure: red bars, *n* = 3 cochleae, 3 mice; after noise exposure: dark red bars, *n* = 3 cochleae, 3 mice; *post hoc* two-sided Student’s *t*-test with no Bonferroni–Holm’s adjustment for multiple testing for medial turn: *p* = 0.306 and midbasal turn: *p* = 0.773). Seven days after noise exposure, larger number of ribbons are observed in Ca_V_1.2^Pax2^ KO mice at all three cochlear turns and are significantly larger at the medial turn (*post hoc* two-sided Student’s *t*-test with no Bonferroni–Holm’s adjustment for multiple testing, *p* = 0.004) and midbasal turn (*post hoc* two-sided Student’s *t*-test with no Bonferroni–Holm’s adjustment for multiple testing, *p* = 0.023) compared to control mice.

Since significant differences in hearing thresholds were observed between Ca_V_1.2^Pax2^ control and KO mice 7–11 days after noise exposure, click-evoked ABR wave amplitude growth functions were compared between control and Ca_V_1.2^Pax2^ KO 7 days after noise exposure for latencies corresponding to the AN (wave I) and SOC (wave III; Melcher et al., 1996). After noise exposure, both ABR wave I (**Figure 6A**, Ca_V_1.2^Pax2^ control: *n* = 5–11 ears, 5–8 mice; Ca_V_1.2^Pax2^ KO: *n* = 6–13 ears, 5–8 mice; two-way ANOVA, *p* = 0.004, *post hoc* one-sided Student’s *t*-test: *p* = 0.038 at 35 dB above threshold, Bonferroni–Holm’s adjustment for multiple testing; *p* = 0.026 at 40 dB above threshold, no Bonferroni–Holm’s adjustment for multiple testing) and ABR wave III amplitudes in Ca_V_1.2^Pax2^ KO mice (**Figure 6B**, Ca_V_1.2^Pax2^ control: *n* = 3–10 ears, 3–6 mice; Ca_V_1.2^Pax2^ KO: *n* = 3–7 ears, 3–4 mice; two-way ANOVA, *p* = 0.013) were less reduced when compared to the controls at high stimulation levels of sound intensity (>30 dB above thresholds).

The changes in the ABR wave I amplitude and the hearing thresholds observed in Ca_V_1.2^Pax2^ mutants were accompanied by an altered number of synaptic ribbons in the three cochlear regions (apical, medial, and midbasal). Ribbon numbers in noise exposed Ca_V_1.2^Pax2^ KO IHCs were slightly different in all cochlear turns 7 days after exposure compared with the ribbon numbers in Ca_V_1.2^Pax2^ KO mice, but were higher in comparison to noise-exposed control mice (**Figure 6C**).

### Ca_V_1.2^Pax2^ KO MICE SHOW REDUCED BDNF LEVELS IN THE COCHLEA

**FIGURE 7:**
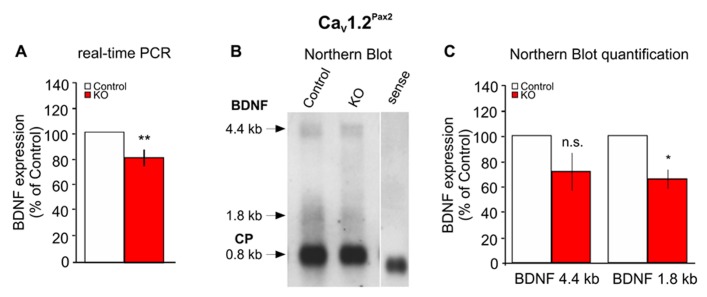
**Reduced expression of BDNF in the cochlea of Ca_V_1.2^Pax2^ KO mice.** **(A)** Real-time PCR of BDNF exon IX mRNA in the cochlea of control (white bar) and Ca_V_1.2^Pax2^ KO mice (red bar), expressed as a percentage of the control set to 100%, shows a significant reduction of the mRNA level in the KO mice (Ca_V_1.2^Pax2^ control: *n* = 11 mice; Ca_V_1.2^Pax2^ KO: *n* = 10 mice, two-sided Student’s *t*-test: *p* = 0.006). **(B)** Northern blot of control and Ca_V_1.2^Pax2^ KO mice cochlea showing the two BDNF isoforms at 1.8 and 4.4 kb and cyclophilin as housekeeping gene at 0.8 kb. **(C)** Quantification of the Northern blot shows a significant reduction of the 1.8 kb BDNF mRNA isoform, but not the 4.4 kb isoform in the cochlea of Ca_V_1.2^Pax2^ KO mice (Ca_V_1.2^Pax2^ control, white bars: *n* = 12 mice; Ca_V_1.2^Pax2^ KO, red bars: *n* = 11 mice, two-sided Student’s *t*-test: *p* = 0.193 for 4.4 kb and *p* = 0.043 for 1.8 kb). Data are expressed as a percentage of control set to 100%.

We showed recently that BDNF controls the number of ribbon synapses in cochlear IHCs and that its absence protects against noise-induced damage (Zuccotti et al., 2012). Most strikingly, L-VGCCs have been described to play a crucial role for activation of nerve growth factor responsiveness in the brain (Aliaga et al., 1998; Shieh et al., 1998; Tabuchi et al., 2000; West et al., 2001; Takeuchi et al., 2002; Tao et al., 2002; Chen et al., 2003). We, therefore, analyzed BDNF levels in the cochleae of Ca_V_1.2^Pax2^ KO mice through real-time PCR and Northern blots using primers or riboprobes spanning the BDNF protein coding exon IX. The Ca_V_1.2^Pax2^ KO mice showed a reduced expression of BDNF mRNA by real-time PCR (**Figure 7A**, Ca_V_1.2^Pax2^ control: *n* = 11 mice; Ca_V_1.2^Pax2^ KO: *n* = 10 mice, two-sided Student’s *t*-test: *p* = 0.006) and Northern blot (**Figures 7B,C**, Ca_V_1.2^Pax2^ control: *n* = 12 mice; Ca_V_1.2^Pax2^ KO: *n* = 11 mice, two-sided Student’s *t*-test: *p* = 0.193 for 4.4 kb and *p* = 0.043 for 1.8 kb), confirming that BDNF expression is regulated by Ca_V_1.2 in the cochlea. The noise protection observed may thus be explained by the reduced BDNF levels when Ca_V_1.2 is deleted in the cochlea, similar to the situation in BDNF^Pax2^ KO mice (Zuccotti et al., 2012).

## DISCUSSION

We recently showed that lack of BDNF hampers IHC synapse physiology and hearing function while it protects against noise-induced hearing loss (NIHL; Zuccotti et al., 2012). In the present study, we provide evidence that part of this crucial BDNF expression may be controlled by the L-VGCC Ca_V_1.2.

Ca_V_1.2 is expressed in SGN and efferent synapses (Waka et al., 2003) that originate in the olivocochlear system in the brainstem and terminate axo-dendritically on afferent type I fibers and axo-somatically on OHCs in the cochlea (White and Warr, 1983). The expression pattern of Ca_V_1.2 is mostly non-overlapping with that of another L-VGCC, Ca_V_1.3, that has been shown to play an essential role for hearing, since its deletion results in deafness (Platzer et al., 2000).

Ca_V_1.2 knock-out mice are lethal at birth and thus, the role of Ca_V_1.2 in hearing function is elusive. Ca_V_1.2 is assumed to participate in altering synapse efficacy through transcriptional control of BDNF (Tabuchi et al., 2000; Zuccotti et al., 2012).

According to West et al. (2001), Ca^2+^ influx through Ca_V_1.2 activates a Ca^2+^ responsive binding protein, CaRF, that acts as a *cis*-acting element on the BDNF exon IV promoter to control BDNF transcription after, e.g., stimulation by kainate. In this study, we analyzed mice with a conditional deletion of Ca_V_1.2 in the cochlea, with Cre recombinase controlled by the Pax2 promoter as recently described for the conditional BDNF KO mice in Zuccotti et al. (2012). We, moreover, compared the effect of deletion of Ca_V_1.2 in the SOC using mice with Cre recombinase controlled by Egr2 promoter (Ca_V_1.2^Egr2^ KO mice).

By measuring ABR thresholds and DPOAE, where the DPOAE reflect the functionality of the OHCs, we found that both Ca_V_1.2^Pax2^ KO and Ca_V_1.2^Egr2^ KO mice do not display significant changes of thresholds and OHC function. This implies that Ca_V_1.2 expression in the cochlea and SOC might not be essential for maintenance of hearing thresholds and OHC function. Furthermore, we analyzed the ABR wave amplitudes, which reflect the number of synchronously firing neurons in distinct structures and nuclei along the ascending auditory pathway (Burkard and Don, 2007). Wave I amplitudes, corresponding to the neural activity from the AN fibers (Johnson and Kiang, 1976), of Ca_V_1.2^Pax2^ KO mice were found significantly reduced starting at 50 dB above threshold compared to the control mice. In contrast, wave III amplitudes, corresponding to the neural activity from the cochlear nucleus (CN) and the SOC in the lower brainstem (Melcher et al., 1996), of the Ca_V_1.2^Pax2^ KO mice are unchanged. Also wave I and III amplitudes of Ca_V_1.2^Egr2^ KO mice appeared normal.

This may indicate that, similar to the role of BDNF (Zuccotti et al., 2012), lack of Ca_V_1.2 in the cochlea hampers the function of the AN, but the phenotype appears to be less pronounced.

*Per contra* to what has been described for the BDNF conditional KO mice (Zuccotti et al., 2012), we could not find significant differences in the number of ribbons in Ca_V_1.2^Pax2^ KO mice. Ribbon structure in IHC maintains a large ready releasable pool in IHCs and defines the reliability within spikes of auditory fibers (Buran et al., 2010). A reduced ABR wave I amplitude was found only at high sound intensities, suggesting that the lack of Ca_V_1.2 in the Ca_V_1.2^Pax2^ KO mice may alter the number of synchronously firing AN fibers that particularly respond to high sound intensities. Indeed, at least two different afferent fiber types exist: high spontaneous firing rate (SR) fibers with greater sensitivities (low-response thresholds) and low SR fibers with less sensitivity (high-response thresholds; Liberman, 1980, 1982). ABR thresholds, depending on the responses of high SR AN fibers, were not affected in Ca_V_1.2^Pax2^ KO mice, while AN responses at high intensities were found to be reduced. Thus, we hypothesize that Ca_V_1.2 in the cochlea might contribute to the neural responses of low SR AN fibers. Future studies may test this hypothesis.

It has been reported that prolonged noise exposure induces various types of hearing damage in rodents, including loss of threshold and disturbance of neural activity along the ascending auditory pathway (Kujawa and Liberman, 2009; Zuccotti et al., 2012; Rüttiger et al., 2013; Singer et al., 2013). Recent studies suggested that the trigger of the loss of SGN in the cochlea, after an acoustic overstimulation, does not originate from IHCs but rather from IHC supporting cells. In particular, inner border and inner phalangeal cells have been proposed to be essential for neuronal survival after IHC damage (Zilberstein et al., 2012). These supporting cell types have also been shown to express BDNF (Sobkowicz et al., 2002; Zuccotti et al., 2012). Around 7–11 days after noise exposure, NIHL and a PTS were observed in both Ca_V_1.2^Pax2^ KO and Ca_V_1.2^Egr2^ KO mouse lines. However, the ABR threshold loss was significantly less pronounced in the Ca_V_1.2^Pax2^ KO mouse mutants than in the Ca_V_1.2^Egr2^ KO mice. This indicates that deletion of Ca_V_1.2 might reduce the vulnerability to acoustic trauma. A reduced vulnerability to acoustic trauma was also observed recently upon deletion of BDNF under the same Pax2 promoter as used here for Ca_V_1.2 deletion (Zuccotti et al., 2012). A first hint that the phenotype of Ca_V_1.2^Pax2^ KO and BDNF^Pax2^ KO after acoustic trauma may be causally related is yielded by the observation that BDNF mRNA is partially reduced in the cochlea of Ca_V_1.2^Pax2^ KO mice. The loss of BDNF mRNA in Ca_V_1.2^Pax2^ KO cochlea mainly affected the short BDNF transcripts. Short and long BDNF transcripts result from alternative 3′ end processing of the BDNF transcripts at 3′ untranslated regions (3′UTRs). Distinct RNA sequences in these BDNF 3′UTRs are reported to differentially regulate neuronal activity changes through altered BDNF stability and translation of these BDNF mRNA isoforms (Lau et al., 2010). While we are far from understanding the role of different BDNF transcripts in the cochlea, we may conclude that Ca_V_1.2 controls short BDNF transcripts. A selected function of Ca_V_1.2 for short BDNF transcripts during its action on destabilization of IHC/afferent contacts during acoustic trauma, but not during BDNF-related retention of the IHC/afferent contacts in the intact system, may be tested in future studies. It has to be taken into consideration that the deletion of Ca_V_1.2 resulted in only 20–40% reduction of BDNF transcripts in the cochlea. This may explain a milder protecting phenotype than that found in the BDNF^Pax2^ KO mice. However, it cannot be ruled out that the protection against the noise-induced damage, when Ca_V_1.2 is deleted in the cochlea, is the result of selective impact on only a subpopulation of cells or the AN fibers.

In conclusion we show that Ca_V_1.2 deletion in the cochlea affects mainly an AN fiber type with high-response thresholds. Moreover, the current data demonstrate that the deletion of Ca_V_1.2 in the cochlea (Ca_V_1.2^Pax2^ mice) rather than the deletion of Ca_V_1.2 in the SOC (Ca_V_1.2^Egr2^ mice) can trigger a reduced vulnerability to acoustic trauma-induced auditory fiber loss. We provide evidence that this effect is linked to a Ca_V_1.2 function on maintaining expression of long or short BDNF transcripts, which in the cochlea may drive destabilization of IHCs/afferent contacts during acoustic trauma as already hypothesized in Zuccotti et al. (2012). This destabilization of IHCs/afferents may originate from a Ca_V_1.2-related BDNF expression and release from supporting cells after cochlear injury. Further studies are on the way to analyze this aspect in more detail.

## Conflict of Interest Statement

The authors declare that the research was conducted in the absence of any commercial or financial relationships that could be construed as a potential conflict of interest.

## References

[B1] Aid T. Kazantseva A. Piirsoo M. Palm K. Timmusk T. (2007). Mouse and rat BDNF gene structure and expression revisited. *J. Neurosci. Res.* 85 525–35 10.1002/jnr.21139 17149751 PMC1878509

[B2] Aliaga E. Rage F. Bustos G. Tapia-Arancibia L. (1998). BDNF gene transcripts in mesencephalic neurons and its differential regulation by NMDA. *Neuroreport* 9 1959–1962 10.1097/00001756-199806220-00008 9674574

[B3] Barg S. Olofsson C. S. Schriever-Abeln J. Wendt A. Gebre-Medhin S. Renström E. (2002). Delay between fusion pore opening and peptide release from large dense-core vesicles in neuroendocrine cells. *Neuron* 33 287–299 11804575 10.1016/s0896-6273(02)00563-9

[B4] Buran B. N. Strenzke N. Neef A. Gundelfinger E. D. Moser T. Liberman M. C. (2010). Onset coding is degraded in auditory nerve fibers from mutant mice lacking synaptic ribbons. *J. Neurosci*. 30 7587–7597 10.1523/JNEUROSCI.0389-10.2010 20519533 PMC2901931

[B5] Burkard R. F. Don M. (2007). “The auditory brainstem response,” in *Auditory Evoked Potentials: Basic Principles and Clinical Application* eds Burkard R. F. Don M. Eggemond J. J. (Baltimore: Lippincott Williams & Wilkins) 229–250

[B6] Catterall W. A. (2000). Structure and regulation of voltage-gated Ca2^+^ channels. *Annu. Rev. Cell Dev. Biol*. 16 521–555 10.1146/annurev.cellbio.16.1.521 11031246

[B7] Chen W. G. West A. E. Tao X. Corfas G. Szentirmay M. N. Sawadogo M. (2003). Upstream stimulatory factors are mediators of Ca2^+^-responsive transcription in neurons. *J. Neurosci.* 23 2572–2581 12684442 10.1523/JNEUROSCI.23-07-02572.2003 PMC6742056

[B8] Coull J. A. Beggs S. Boudreau D. Boivin D. Tsuda M. Inoue K. (2005). BDNF from microglia causes the shift in neuronal anion gradient underlying neuropathic pain. *Nature* 438 1017–1021 10.1038/nature04223 16355225

[B9] Davare M. A. Dong F. Rubin C. S. Hell J. W. (1999). The A-kinase anchor protein MAP2B and cAMP-dependent protein kinase are associated with class C L-type calcium channels in neurons. *J. Biol. Chem*. 274 30280–30287 10.1074/jbc.274.42.30280 10514522

[B10] Dugich-Djordjevic M. M. Tocco G. Lapchak P. A. Pasinetti G. M. Najm I. Baudry M. (1992). Regionally specific and rapid increases in brain-derived neurotrophic factor messenger RNA in the adult rat brain following seizures induced by systemic administration of kainic acid. *Neuroscience* 47 303–315 10.1016/0306-4522(92)90246-X 1641125

[B11] El-Badry M. M. McFadden S. L. (2007). Electrophysiological correlates of progressive sensorineural pathology in carboplatin-treated chinchillas. *Brain Res.* 1134 122–130 10.1016/j.brainres.2006.11.078 17198689 PMC1817725

[B12] Engel J. Braig C. Rüttiger L. Kuhn S. Zimmermann U. Blin N. (2006). Two classes of outer hair cells along the tonotopic axis of the cochlea. *Neuroscience* 143 837–849 17074442 10.1016/j.neuroscience.2006.08.060

[B13] Favereaux A. Thoumine O. Bouali-Benazzouz R. Roques V. Papon M.-A. Salam S. A. (2011). Bidirectional integrative regulation of Ca_V_1.2 calcium channel by microRNA miR-103: role in pain. *EMBO J.* 30 3830–3841 10.1038/emboj.2011.249 21804529 PMC3173784

[B14] Feliciano M. Potashner S. J. (1995). Evidence for a glutamatergic pathway from the guinea pig auditory cortex to the inferior colliculus. *J. Neurochem.* 65 1348–1357 10.1046/j.1471-4159.1995.65031348.x 7643112

[B15] Fuchs P. A. (2005). Time and intensity coding at the hair cell’s ribbon synapse. *J. Physiol.* 566 7–12 10.1113/jphysiol.2004.082214 15845587 PMC1464726

[B16] Green G. E. Khan K. M. Beisel D. W. Drescher M. J. Hatfield J. S. Drescher D. G. (1996). Calcium channel subunits in the mouse cochlea. *J. Neurochem.* 67 37–45 10.1046/j.1471-4159.1996.67010037.x 8667015

[B17] Hall J. Thomas K. L. Everitt B. J. (2000). Rapid and selective induction of BDNF expression in the hippocampus during contextual learning. *Nat. Neurosci.* 3 533–535 10.1038/75698 10816306

[B18] Hell J. W. Westenbroek R. E. Warner C. Ahlijanian M. K. Prystay W. Gilbert M. (1993). Identification and differential subcellular localization of the neuronal class C and class D L-type calcium channel alpha 1 subunits. *J. Cell Biol.* 123 949–962 10.1083/jcb.123.4.949 8227151 PMC2200142

[B19] Johnson D. H. Kiang N. Y. (1976). Analysis of discharges recorded simultaneously from pairs of auditory nerve fibers. *Biophys. J.* 16 719–734 10.1016/S0006-3495(76)85724-4 938715 PMC1334896

[B20] Knipper M. Gestwa L. Ten Cate W. J. Lautermann J. Brugger H. Maier H. (1999). Distinct thyroid hormone-dependent expression of TrKB and p75NGFR in nonneuronal cells during the critical TH-dependent period of the cochlea. *J. Neurobiol.* 38 338–356 10.1002/(SICI)1097-4695(19990215)38:3 10022577

[B21] Kolarow R. Brigadski T. Lessmann V. (2007). Postsynaptic secretion of BDNF and NT-3 from hippocampal neurons depends on calcium calmodulin kinase II signaling and proceeds via delayed fusion pore opening. *J. Neurosci.* 27 10350–10364 10.1523/JNEUROSCI.0692-07.2007 17898207 PMC6673152

[B22] Kujawa S. G. Liberman M. C. (2009). Adding insult to injury: cochlear nerve degeneration after “temporary” noise-induced hearing loss. *J. Neurosci.* 29 14077–14085 10.1523/JNEUROSCI.2845-09.2009 19906956 PMC2812055

[B23] Lau A. G. Irier H. A. Gu J. Tian D. Ku L. Liu G. (2010). Distinct 3′UTRs differentially regulate activity-dependent translation of brain-derived neurotrophic factor (BDNF). *Proc. Natl. Acad. Sci. U.S.A.* 107 15945–15950 10.1073/pnas.1002929107 20733072 PMC2936648

[B24] Liberman M. C. (1980). Morphological differences among radial afferent fibers in the cat cochlea: an electron-microscopic study of serial sections. *Hear. Res.* 3 45–63 10.1016/0378-5955(80)90007-6 7400048

[B25] Liberman M. C. (1982). The cochlear frequency map for the cat: labeling auditory-nerve fibers of known characteristic frequency. *J. Acoust. Soc. Am.* 72 1441–1449 10.1121/1.388677 7175031

[B26] Lin H. W. Furman A. C. Kujawa S. G. Liberman M. C. (2011). Primary neural degeneration in the Guinea pig cochlea after reversible noise-induced threshold shift. *J. Assoc. Res. Otolaryngol.* 12 605–616 10.1007/s10162-011-0277-0 21688060 PMC3173555

[B27] Lubin F. D. Roth T. L. Sweatt J. D. (2008). Epigenetic regulation of BDNF gene transcription in the consolidation of fear memory. *J. Neurosci.* 28 10576–10586 10.1523/JNEUROSCI.1786-08.2008 18923034 PMC3312036

[B28] Matthews G. Fuchs P. (2010). The diverse roles of ribbon synapses in sensory neurotransmission. *Nat. Rev. Neurosci.* 11 812–22 10.1038/nrn2924 21045860 PMC3065184

[B29] Melcher J. R. Guinan J. J. Knudson I. M. Kiang N. Y. (1996). Generators of the brainstem auditory evoked potential in cat. II. Correlating lesion sites with waveform changes. *Hear. Res.* 93 28–51 10.1016/0378-5955(95)00179-4 8735067

[B30] Mo W. Nicolson T. (2011). Both pre- and postsynaptic activity of Nsf prevents degeneration of hair-cell synapses. *PLoS ONE* 6:e27146 10.1371/journal.pone.0027146 PMC3207842 22073277

[B31] Moser T. Brandt A. Lysakowski A. (2006). Hair cell ribbon synapses. *Cell Tissue Res.* 326 347–59 10.1007/s00441-006-0276-3 16944206 PMC4142044

[B32] Müller M. (1991). Frequency representation in the rat cochlea. *Hear. Res.* 51 247–254 2032960 10.1016/0378-5955(91)90041-7

[B33] Neeper S. A. Gómez-Pinilla F. Choi J. Cotman C. (1995). Exercise and brain neurotrophins. *Nature* 373 109 10.1038/373109a0 7816089

[B34] Ohyama T. Groves A. K. (2004). Generation of Pax2-Cre mice by modification of a Pax2 bacterial artificial chromosome. *Genesis* 38 195–199 10.1002/gene.20017 15083520

[B35] Platzer J. Engel J. Schrott-Fischer A. Stephan K. Bova S. Chen H. (2000). congenital deafness and sinoatrial node dysfunction in mice lacking class D L-type Ca2^+^ channels. *Cell* 102 89–97 10.1016/S0092-8674(00)00013-1 10929716

[B36] Rosengauer E. Hartwich H. Hartmann A. M. Rudnicki A. Satheesh S. V. Avraham K. B. (2012). Egr2::cre mediated conditional ablation of dicer disrupts histogenesis of mammalian central auditory nuclei. *PLoS ONE* 7:e49503 10.1371/journal.pone.0049503 PMC3495878 23152916

[B37] Rüttiger L. Singer W. Panford-Walsh R. Matsumoto M. Lee S. C. Zuccotti A. (2013). The reduced cochlear output and the failure to adapt the central auditory response causes tinnitus in noise exposed rats. *PLoS ONE* 8:e57247 10.1371/journal.pone.0057247 PMC3596376 23516401

[B38] Salvi R. J. Henderson D. Hamernik R. P. Colletti V. (eds). (1986). *Basic and Applied Aspects of Noise-Induced Hearing Loss*. Boston, MA: Springer

[B39] Satheesh S. V. Kunert K. Rüttiger L. Zuccotti A. Schönig K. Friauf E. (2012). Retrocochlear function of the peripheral deafness gene Cacna1d. *Hum. Mol. Genet.* 21 3896–3909 10.1093/hmg/dds217 22678062

[B40] Schimmang T. Tan J. Müller M. Zimmermann U. Rohbock K. Kôpschall I. (2003). Lack of BDNF and TrkB signalling in the postnatal cochlea leads to a spatial reshaping of innervation along the tonotopic axis and hearing loss. *Development* 130 4741–4750 12925599 10.1242/dev.00676

[B41] Schmitz F. (2009). The making of synaptic ribbons: how they are built and what they do. *Neuroscientist* 15 611–624 10.1177/1073858409340253 19700740

[B42] Schug N. Braig C. Zimmermann U. Engel J. Winter H. Ruth P. (2006). Differential expression of otoferlin in brain, vestibular system, immature and mature cochlea of the rat. *Eur. J. Neurosci.* 24 3372–3380 10.1111/j.1460-9568.2006.05225.x 17229086

[B43] Seisenberger C. Specht V. Welling A. Platzer J. Pfeifer A. Kühbandner S. (2000). Functional embryonic cardiomyocytes after disruption of the L-type alpha1C (Cav1.2) calcium channel gene in the mouse. *J. Biol. Chem.* 275 39193–39199 10.1074/jbc.M006467200 10973973

[B44] Shieh P. B. Hu S. C. Bobb K. Timmusk T. Ghosh A. (1998). Identification of a signaling pathway involved in calcium regulation of BDNF expression. *Neuron* 20 727–740 10.1016/S0896-6273(00)81011-9 9581764

[B45] Singer W. Panford-Walsh R. Watermann D. Hendrich O. Zimmermann U. Köpschall I. (2008). Salicylate alters the expression of calcium response transcription factor 1 in the cochlea: implications for brain-derived neurotrophic factor transcriptional regulation. *Mol. Pharmacol.* 73 1085–1091 10.1124/mol.107.041814 18198284

[B46] Singer W. Zuccotti A. Jaumann M. Lee S. C. Panford-Walsh R. Xiong H. (2013). Noise-induced inner hair cell ribbon loss disturbs central arc mobilization: a novel molecular paradigm for understanding tinnitus. *Mol. Neurobiol.* 47 261–279 10.1007/s12035-012-8372-8 23154938

[B47] Sobkowicz H. M. August B. K. Slapnick S. M. (2002). Influence of neurotrophins on the synaptogenesis of inner hair cells in the deaf Bronx waltzer (bv) mouse organ of Corti in culture. *Int. J. Dev. Neurosci.* 20 537–554 10.1016/S0736-5748(02)00084-9 12485622

[B48] Tabuchi A. Nakaoka R. Amano K. Yukimine M. Andoh T. Kuraishi Y. (2000). Differential activation of brain-derived neurotrophic factor gene promoters I and III by Ca2^+^ signals evoked via L-type voltage-dependent and *N*-methyl-D-aspartate receptor Ca2^+^ channels. *J. Biol. Chem.* 275 17269–17275 10.1074/jbc.M909538199 10748141

[B49] Takeuchi Y. Miyamoto E. Fukunaga K. (2002). Analysis on the promoter region of exon IV brain-derived neurotrophic factor in NG108-15 cells. *J. Neurochem.* 83 67–79 10.1046/j.1471-4159.2002.01096.x 12358730

[B50] Tan J. Rüttiger L. Panford-Walsh R. Singer W. Schulze H. Kilian S. B. (2007). Tinnitus behavior and hearing function correlate with the reciprocal expression patterns of BDNF and Arg3.1/arc in auditory neurons following acoustic trauma. *Neuroscience* 145 715–726 17275194 10.1016/j.neuroscience.2006.11.067

[B51] Tao X. West A. E. Chen W. G. Corfas G. Greenberg M. E. (2002). A calcium-responsive transcription factor, CaRF, that regulates neuronal activity-dependent expression of BDNF. *Neuron* 33 383–395 10.1016/S0896-6273(01)00561-X 11832226

[B52] Trang T. Beggs S. Salter M. W. (2011). Brain-derived neurotrophic factor from microglia: a molecular substrate for neuropathic pain. *Neuron Glia Biol.* 7 99–108 10.1017/S1740925X12000087 22613083 PMC3748035

[B53] Voiculescu O. Charnay P. Schneider-Maunoury S. (2000). Expression pattern of a Krox-20/Cre knock-in allele in the developing hindbrain, bones, and peripheral nervous system. *Genesis* 26 123–126 10.1002/(SICI)1526-968X(200002)26:2 10686605

[B54] Waka N. Knipper M. Engel J. (2003). Localization of the calcium channel subunits Cav1.2 (alpha1C) and Cav2.3 (alpha1E) in the mouse organ of Corti. *Histol. Histopathol.* 18 1115–1123 12973680 10.14670/HH-18.1115

[B55] Warr W. B. Guinan J. J. (1979). Efferent innervation of the organ of corti: two separate systems. *Brain Res.* 173 152–155 10.1016/0006-8993(79)91104-1 487078

[B56] West A. E. Chen W. G. Dalva M. B. Dolmetsch R. E. Kornhauser J. M. Shaywitz A. J. (2001). Calcium regulation of neuronal gene expression. *Proc. Natl. Acad. Sci. U.S.A.* 98 11024–11031 10.1073/pnas.191352298 11572963 PMC58677

[B57] White J. S. Warr W. B. (1983). The dual origins of the olivocochlear bundle in the albino rat. *J. Comp. Neurol.* 219 203–214 10.1002/cne.902190206 6619338

[B58] Xiao Z. Suga N. (2002). Modulation of cochlear hair cells by the auditory cortex in the mustached bat. *Nat. Neurosci.* 5 57–63 10.1038/nn786 11753417

[B59] Zampini V. Johnson S. L. Franz C. Lawrence N. D. Münkner S. Engel J. (2010). Elementary properties of Ca_V_1.3 Ca(2^+^) channels expressed in mouse cochlear inner hair cells. *J. Physiol.* 588 187–199 10.1113/jphysiol.2009.181917 19917569 PMC2817446

[B60] Zheng F. Zhou X. Luo Y. Xiao H. Wayman G. Wang H. (2011). Regulation of brain-derived neurotrophic factor exon IV transcription through calcium responsive elements in cortical neurons. *PLoS ONE* 6:e28441 10.1371/journal.pone.0028441 PMC3235121 22174809

[B61] Zilberstein Y. Liberman M. C. Corfas G. (2012). Inner hair cells are not required for survival of spiral ganglion neurons in the adult cochlea. *J. Neurosci.* 32 405–410 10.1523/JNEUROSCI.4678-11.2012 22238076 PMC3678770

[B62] Zuccotti A. Clementi S. Reinbothe T. Torrente A. Vandael D. H. Pirone A. (2011). Structural and functional differences between L-type calcium channels: crucial issues for future selective targeting. *Trends Pharmacol. Sci.* 32 366–375 10.1016/j.tips.2011.02.012 21450352

[B63] Zuccotti A. Kuhn S. Johnson S. L. Franz C. Singer W. Hecker D. (2012). Lack of brain-derived neurotrophic factor hampers inner hair cell synapse physiology, but protects against noise-induced hearing loss. *J. Neurosci.* 32 8545–8553 10.1523/JNEUROSCI.1247-12.2012 22723694 PMC6620992

